# Bacterial microbiota of Kazakhstan cheese revealed by single molecule real time (SMRT) sequencing and its comparison with Belgian, Kalmykian and Italian artisanal cheeses

**DOI:** 10.1186/s12866-016-0911-4

**Published:** 2017-01-09

**Authors:** Jing Li, Yi Zheng, Haiyan Xu, Xiaoxia Xi, Qiangchuan Hou, Shuzhen Feng, Laga Wuri, Yanfei Bian, Zhongjie Yu, Lai-Yu Kwok, Zhihong Sun, Tiansong Sun

**Affiliations:** Key Laboratory of Dairy Biotechnology and Engineering, Inner Mongolia Agricultural University, Hohhot, 010018 People’s Republic of China

**Keywords:** Single molecule, Real-time (SMRT), Cheeses, Bacterial diversity, Kazakhstan

## Abstract

**Background:**

In Kazakhstan, traditional artisanal cheeses have a long history and are widely consumed. The unique characteristics of local artisanal cheeses are almost completely preserved. However, their microbial communities have rarely been reported. The current study firstly generated the Single Molecule, Real-Time (SMRT) sequencing bacterial diversity profiles of 6 traditional artisanal cheese samples of Kazakhstan origin, followed by comparatively analyzed the microbiota composition between the current dataset and those from cheeses originated from Belgium, Russian Republic of Kalmykia (Kalmykia) and Italy.

**Results:**

Across the Kazakhstan cheese samples, a total of 238 bacterial species belonging to 14 phyla and 140 genera were identified. *Lactococcus lactis* (28.93%), *Lactobacillus helveticus* (26.43%), *Streptococcus thermophilus* (12.18%) and *Lactobacillus delbrueckii* (12.15%) were the dominant bacterial species for these samples. To further evaluate the cheese bacterial diversity of Kazakhstan cheeses in comparison with those from other geographic origins, 16S rRNA datasets of 36 artisanal cheeses from Belgium, Russian Republic of Kalmykia (Kalmykia) and Italy were retrieved from public databases. The cheese bacterial microbiota communities were largely different across sample origins. By principal coordinate analysis (PCoA) and multivariate analysis of variance (MANOVA), the structure of the Kazakhstan artisanal cheese samples was found to be different from those of the other geographic origins. Furthermore, the redundancy analysis (RDA) identified 16 bacterial OTUs as the key variables responsible for such microbiota structural difference.

**Conclusion:**

Our results together suggest that the diversity of bacterial communities in different groups is stratified by geographic region. This study does not only provide novel information on the bacterial microbiota of traditional artisanal cheese of Kazakhstan at species level, but also interesting insights into the bacterial diversity of artisanal cheeses of various geographical origins.

**Electronic supplementary material:**

The online version of this article (doi:10.1186/s12866-016-0911-4) contains supplementary material, which is available to authorized users.

## Background

Kazakhstan is a multi-ethnic state and livestock-based country [1] that locates in the European and Asian continents; and the country has a long history since the Neolithic Age [2]. Artisanal foods are popular with the local area and the processing technology of such traditional food is almost completely preserved. Cheeses, as one of the oldest fermented foods, have a long history from approximately the early Bronze Age [3, 4]. Moreover, the classical Kazakhstan diet comprises a large proportion of local dairy products [5], so the cheese processing technology is traditional. They are often produced by individual households or small regional factories; and they are made in a small scale with naturally occurring bacteria [6]. The traditional Kazakhstan cheeses are produced through various steps: firstly, fresh raw cow milk is collected, followed by pasteurization. The natural whey is then added to the pasteurized milk to enhance coagulation under natural conditions. Then, the whey is drained through heating and extrusion. The curds without ripening are shaped into different forms by using different molds [7]. The starter cultures for making Kazakhstan artisanal cheeses are mainly natural whey. Thus the natural microbial communities of the resultant cheeses are very rich and complex. To sum up, the artisanal cheese of Kazakhstan has its unique characteristics.

The natural microbial communities play an important role in these artisanal cheeses; and they contribute significantly to the cheese quality and properties like flavor, texture and appearance [8]. Meanwhile, it is known that cheeses and cheese-derived microorganisms can directly and/or indirectly influence the host microbiota, systemic immune responses and overall health [9–12]. Therefore, understanding the intrinsic composition of bacterial community is of high scientific interest. High-throughput sequencing has become a common technique that provides accurate depiction of the microbial communities present in traditional artisanal cheeses. Based on this approach, Quigley et al. for the first time detected the presence of several genera in artisanal cheeses, including *Arthrobacter, Brachybacterium, Faecalibacterium, Prevotella,* and *Helcococcus*, which confirmed that the level of cheese maturation had an influence on the *Lactobacillus* population [13]. The bacterial communities of some specific cheese types have been studied, such as mozzarella cheese [14], Croatian cheese [15], Herve cheese [16], and Mexican Poro cheese [17]. However, the characteristics of the microbial communities in Kazakhstan cheese have rarely been reported.

The third generation PacBio single molecule, real-time (SMRT) sequencing technology, a new and advanced high-throughput sequencing tool, generates long reads and allows high taxonomic resolution to the genus and even species level when coupled to full length 16S rRNA gene sequencing [18]. It has been successfully applied in the evaluation of milk bacterial contamination [19]. Thus, the aim of this study was to provide high resolution bacterial microbiota profiles of Kazakhstan artisanal cheese samples using the PacBio SMRT platform; a second aim of our study was to comparatively analyze the bacterial microbiota of cheese from different regions, including Kazakhstan, Belgium, Russian Republic of Kalmykia (Kalmykia) and Italy. To do so, 36 16S rRNA gene datasets were retrieved from public databases. Our study provides novel information on the microbial communities of Kazakhstan traditional cheese products.

## Methods

### Sample information

A total of six traditional artisanal cheeses were collected from two different artisanal factories of Kazakhstan (K1-K4 and K5-K6 collected respectively from Alma-Ata and Jambyl provinces, Additional file 1: Figure S1). The manufacturing process of these cheeses was similar, as described previously. Samples were collected aseptically and were stored in vacuum bags as soon as they were sampled. They were kept cold while being transported to the laboratory. The nutritional information of these cheeses is provided by the factories (Additional file 2: Table S1).

Apart from the 6 cheese samples collected from Kazakhstan. Datasets of 16S rRNA gene fragments of 36 artisanal cheese samples [14, 16] from Belgium, Russian Republic of Kalmykia (Kalmykia) and Italy were extracted from public databases for comparative analysis. The information of the samples is provided in Table 1.

**Table 1:** Sample information

Sample	Sampling location	Country	Milk source	Starter	Sequenced 16S rRNA region	Sequencing technology	Cheese production process	Accession number
K1- K4	Alma-Ata Province	Kazakhstan	Cow milk	Natural whey culture	Full length	SMRT technology, Pacific Biosciences	The fresh raw cow milk is pasteurized. The natural whey is added to the pasteurized milk and allows coagulating. Then the whey is drained through heating and extrusion. The curds (without ripening) are shaped into different forms by different molds [7].	BioProject ID: PRJNA347428
K5-K6	Jambyl Province							
B1- B22	Herve	Belgium	Cow milk	Not given	V1-V3 regions	GS Junior platform, 454 Life Sciences	Milk is supplemented with rennet. After coagulation, milk curds are cut in to small pieces like the size of a hazelnut, before being poured into square-shaped molds. The molds are turned over every few hours for 2 days. During the ripening period, cheese is washed 2 or 3 times per week with salt water or pure salts to enhance smear formation [16].	BioProject ID PRJNA238292 [16]
R1-R6	Yashkul	Russian Republic of Kalmykia	Cow milk	Natural whey culture	V1-V3 regions	FLX454 Titanium System, 454 Life Sciences	Fresh raw cow milk is supplemented with natural whey cultures as starters. After curding, the whey is drained through heating and extrusion. The curds without ripening are shaped by different mold	MG-RAST ID No. 4682839.3 - 4682844.3
I1-I6	Campania region	Italy	Buffalo milk	Natural whey culture	V1-V3 regions	GS Junior platform, 454 Life Sciences	Raw milk is heated at 37 °C. Then, natural whey cultures are added. After a curd-ripening phase (4.0–4.5 h at 35–37 °C), the curd is drained. The drained curd is stretched in hot water (90–95 °C) before being hand-molded to get the typical round shape [66].	Sequence Read Archive project SRP014821 [14]

### DNA extraction and PCR amplification

Total sample DNA was extracted by a combination of described methods [20–22] with slight modifications. Cheese samples were collected from different parts of the same cheese. Then, cheese samples were mixed together and crushed to uniform powders. Two grams of cheese powders were suspended in 2 mL TE buffer (10 mM Tris · Cl, 1 mM EDTA, pH8.0). The mixture was centrifuged at 8,000 × g for 5 min. Then the pellets were washed with 500 μL TE buffer at 8,000 × g for 5 min in clean 1.5 mL microcentrifuge tubes. Washed cell pellets were resuspended in 500 μL TE buffer. Suspension was frozen for 2 min by liquid nitrogen, before being incubated at 65 °Cfor 3 min; the above steps were repeated 3 times. Proteinase K solution (15 μL, 20 mg /mL in TE, Amrescolnc., USA) and 60 μL SDS solution (10%) were added, mixed and incubated at 37 °C, 300 rpm/min overnight. The mixture was incubated with 100 μL NaCl (5 M) and 80 μL CTAB solutions (10% cetyltrimethylammonium bromide, 0.7 M NaCl) at 65 °C for 30 min and extracted with 1 vol. phenol/chloroform/isoamylalcohol. After centrifugation, the supernatant was re-extracted with chloroform/ isoamylalcohol (1:1 ratio). After another centrifugation, DNA was obtained by the addition of 500 μL isopropanol and 100 μL NaAC (0.5 M) at −20 °C for 20 min. The pellets were washed in 500 μl ethanol (70%) and dried. Then the DNA was dissolved in 50–150 μL TE at 37 °C for 1 h and added with 100 μL RNAase solution (100 μg/mL in TE, Sigma-Aldrich, USA). The quality of DNA was checked in 0.8% agarose gel electrophoresis (Liuyi Biotechnology, China) and spectrophotometer (Thermo Scientific, USA). All extracted DNA samples were stored at −20 °C for further analysis.

The forward 27 F (5’-GAGAGTTTGATCCTGGCTCAG-3’) and the reverse 1541R (5’- AAGGAGGTGATCCAGCCGCA-3’) primers [23] were used to amplify the 16S rRNA gene fragments with PCRBIO *Taq* DNA polymerase (PCR Biosystems Ltd., UK). The volume of the final reaction mixture was 50 μL containing 10 μL 5× PCRBIO reaction buffer, 2 μL forward primer (10 μM), 2 μL reverse primer (10 μM), 1 μL template DNA, 1 μL PCRBIO *Taq* DNA polymerase (5 units/μL) and 34 μL double distilled H_2_O. Conditions of PCR amplification was an initial denaturing step for 5 min at 95 °C, followed by 30 cycles of a denaturing step at 95 °C for 30 s, primer annealing at 58 °C for 45 s and elongation at 72 °C for 1 min. The program was completed with a final elongation step at 72 °C for 7 min. The PCR products were purified according to the protocol of the Pacific Biosciences (http://www.pacb.com/wp-content/uploads/2015/09/Procedure-Checklist-2-kb-Template-Preparation-and-Sequencing.pdf). The quality of PCR products was checked by using an Agilent DNA 1000 Kit and an Agilent 2100 Bioanalyser (Agilent Technologies, USA) according to the manufacturer’s instructions.

### Single molecule real-time sequencing of Kazakhstan cheese 16S rRNA

The amplicons of the 16S rDNA regions purified were used to construct the DNA libraries using the Pacific Biosciences Template Prep Kit 2.0. Amplicons of the16S rDNA regions described previously were sequenced using P6/C4 chemistry on a PacBio RS II instrument (Pacific Biosciences, USA). The quality control for PCR amplifications and sequence preprocessing was performed by the methods described previously by Mosher et al., 2013 [24]. Raw data were processed by the protocol RS_ReadsOfinsert.1. Raw reads were first filtered according to the following criteria restrictively: (i) minimum full passes was up to 5; (ii) minimum predicted accuracy was 90; (iii) 1400 was the minimum read length of inserts, and (iv) 1800 was the maximum read length.

### Bioinformatics processing and statistical analyses

According to the barcode, the extracted high-quality sequences obtained by SMRT technology were sorted. Then the barcodes and primer were removed from the extracted high-quality sequences - dataset A. Datasets of 16S rRNA gene fragments of 36 artisanal cheese samples from Belgium, Kalmykia and Italy were also first filtered, with the selection for sequence read length of over 425 bp to build the dataset B. Sequences from dataset A and dataset B were then analyzed by using QIIME 1.7 (Quantitative Insights into Microbial Ecology) software. The sequences were aligned using PyNAST [25] and UCLUST [26] under 100% clustering of sequence identity to obtain representative sequences. The resultant sequences were clustered to obtain operational taxonomic units (OTU) under the threshold of 98.65% identity [27] by the method of the UCLUST algorithm. ChimeraSlayer [28] was used to remove the chimeric sequences in the representative set of OTUs. The taxonomy of each bacterial OTU representative sequence was assigned with the Ribosomal Database Project (RDP) database (version 11.4) [29] and Greengenes (version 13_8) [30] using classifier with an 80% confidence threshold. A representative chimera-checked OTU set was applied to construct a *denovo* taxonomic tree in FastTree for downstream analysis [31]. The Shannon-Wiener index, Simpson’s diversity, Chao1 and rarefaction estimators were employed to measure the sequencing diversity. The weighted and unweighted principal coordinate analyses (PCoA) based on UniFrac metrics [32] were performed to assess the microbiota structure of different samples.

The diversity of bacterial communities of the 40 artisanal cheeses across 4 countries was analyzed by analysis of variance (ANOVA) based on UniFrac distances as calculated by the SAS software version 9.2 (SAS Institute Inc., Cary, NC) [33]. Multivariate ANOVA (MANOVA) and clustering of microbiota of cheeses based on Mahalanobis distances [34] were conducted by the Matlab R2011b software (the MathWorks, Natick, MA, USA). Differences in the bacterial populations between samples were evaluated with Kruskal-Wallis tests [35]. Redundancy analysis (RDA) was applied to identify the key microbial groups that contributed to the structural difference with the software Canoco for Windows 4.5 (Microcomputer Power, NY, USA). The graph presentations were generated by the R package version 3.1.2 (https://www.r-project.org/) and the Origin software version 8.5 (OriginLab Corporation, Hampton, MA).

## Results

### Richness and diversity analysis of bacterial community composition of Kazakhstan cheese

In this study, the bacterial diversity profiles of six traditional artisanal cheeses were obtained by SMRT sequencing technology. A total of 30884 of bacterial 16S rRNA raw reads were obtained. An average of 5147.33 sequences (SD = 2647.59) were generated for each sample. The number of reads and assigned OTUs of the six samples are shown in Additional file 2: Table S2. After alignment and sequence identity clustering, a total of 8875 unique OTUs were obtained. The average representative OTUs for each individual sample was 1479.17.

Additionally, the results of the Shannon index, Simpson index, Chao1 index, and the number of observed species are shown in Additional file 2: Table S2. The diversity indexes, including Chao 1 index (ranged from 893.90–8569.60), the Shannon index (4.72–8.75), Simpson index (0.84–0.99) and the number of observed species (315.89–1214.52), show that the bacterial communities varied apparently among the six cheese samples. The bacterial diversity of sample K6 was lowest compared to other samples. The Shannon diversity and the rarefaction curves indicate that the overall bacterial diversity was well captured and represented (Additional file 3: Figure S2).

### Bacterial composition in the Kazakhstan cheese samples

Each bacterial OTU representative sequence was taxonomically assigned with the Greengenes (version 13.8) and the Ribosomal Database Project (RDP) II database (version 11.4). A total of 14 phyla were identified in the sampled cheeses (Additional file 2: Table S3). The major bacterial phyla (with mean relative abundance of >0.1%) were Firmicutes (92.47%), Proteobacteria (6.95%), Actinobacteria (0.24%), and Cyanobacteria (0.17%).

**Fig. 1:**
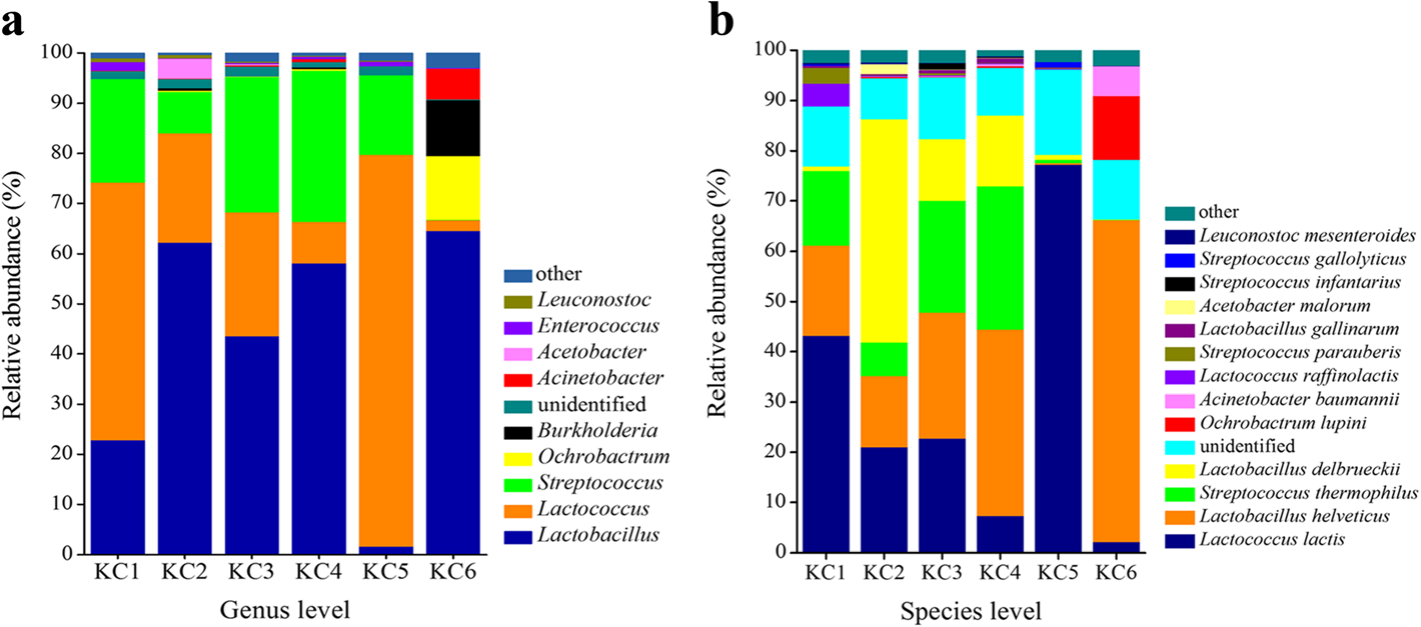
Relative abundances and bacterial diversity at genus (**a**) and species (**b**) levels

At genus level, 140 bacterial genera were identified (Fig. 1a). The major genera (average relative abundance of >1%) detected in the samples included *Lactobacillus* (42.12%), *Lactococcus* (31.07%), *Streptococcus* (16.99%), *Ochrobactrum* (2.25%) and *Burkholderia* (1.98%). Large variations in bacterial composition existed among samples. *Lactobacillus* was the predominant bacterial genus of the samples K1-K4, and K6. The prevalent bacterial genus of the sample K5 was *Lactococcus*. The proportions of *Lactobacillus* and *Lactococcus* in Kazakhstan cheeses ranged from 1.53–64.51% and 2.08–78.17%, respectively.

From the six cheese samples, a total of 238 different species were detected. *Lactococcus lactis* (28.93%), *Lactobacillus helveticus* (26.43%), *Streptococcus thermophilus* (12.18%) and *Lactobacillus delbrueckii* (12.15%) were the dominant species. *Ochrobactrum lupine* (2.23%) and *Acinetobacter baumannii* (1.09%) were detected across cheese samples with varying abundances (Fig. 1b). *Lactobacillus helveticus* was dominating in samples K3, K4 and K6, contributing to 24.98%, 37.11% and 64.05% of the bacterial population, respectively. *Lactococcus lactis* (43.18%) was the predominant species of sample K1. The relative abundance of *L. delbrueckii* in sample K6 was highest among all samples. The relative abundances of *Ochrobactrum lupine* and *Acinetobacter baumannii* of the six samples ranged from 0.06–12.75% and from 0.05–5.85%, respectively. For sample K6, the relative abundances of *Ochrobactrum lupine* and *Acinetobacter baumannii* represented 12.75% and 5.85% of the total bacterial sequences.

### Comparison of bacterial profiles of artisanal cheese in different locations

Datasets of 16S rRNA gene fragments of three kinds of cheeses, respectively from Belgium, Russian Republic of Kalmykia (Kalmykia) and Italy were used for the comparative analysis together with that of Kazakhstan cheese generated from the current study.

**Fig. 2:**
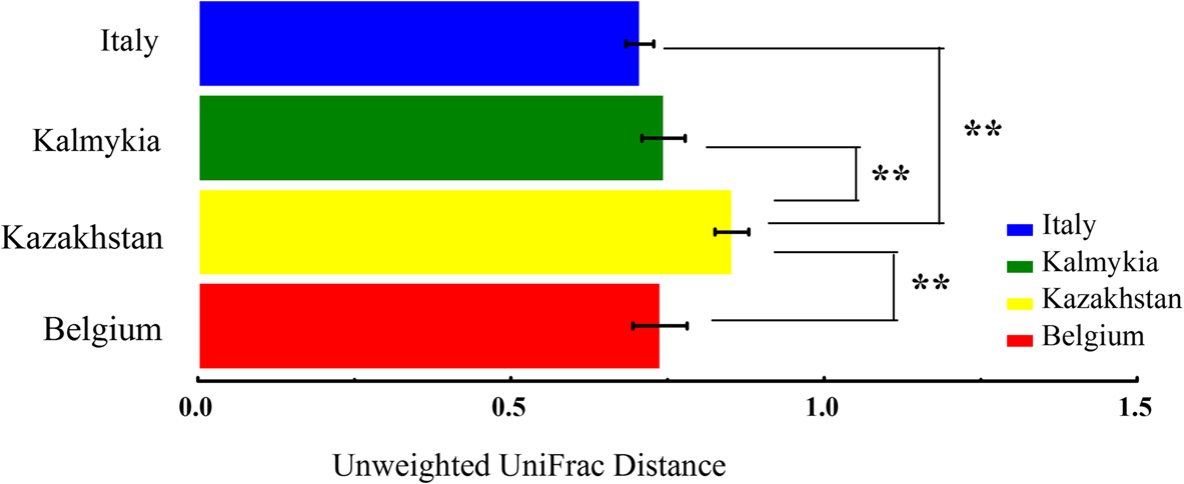
Comparison of the unweighted UniFrac distance of cheese bacterial communities from four countries “**” represents *p* < 0.01

The average UniFrac distances of the four groups were assessed with ANOVA to evaluate the differences among the samples (Fig. 2). The unweighted UniFrac distance of the Kazakhstan cheeses was significantly different from other groups (*p* < 0.01), suggesting that the Kazakhstan cheese had some unique features.

**Fig. 3:**
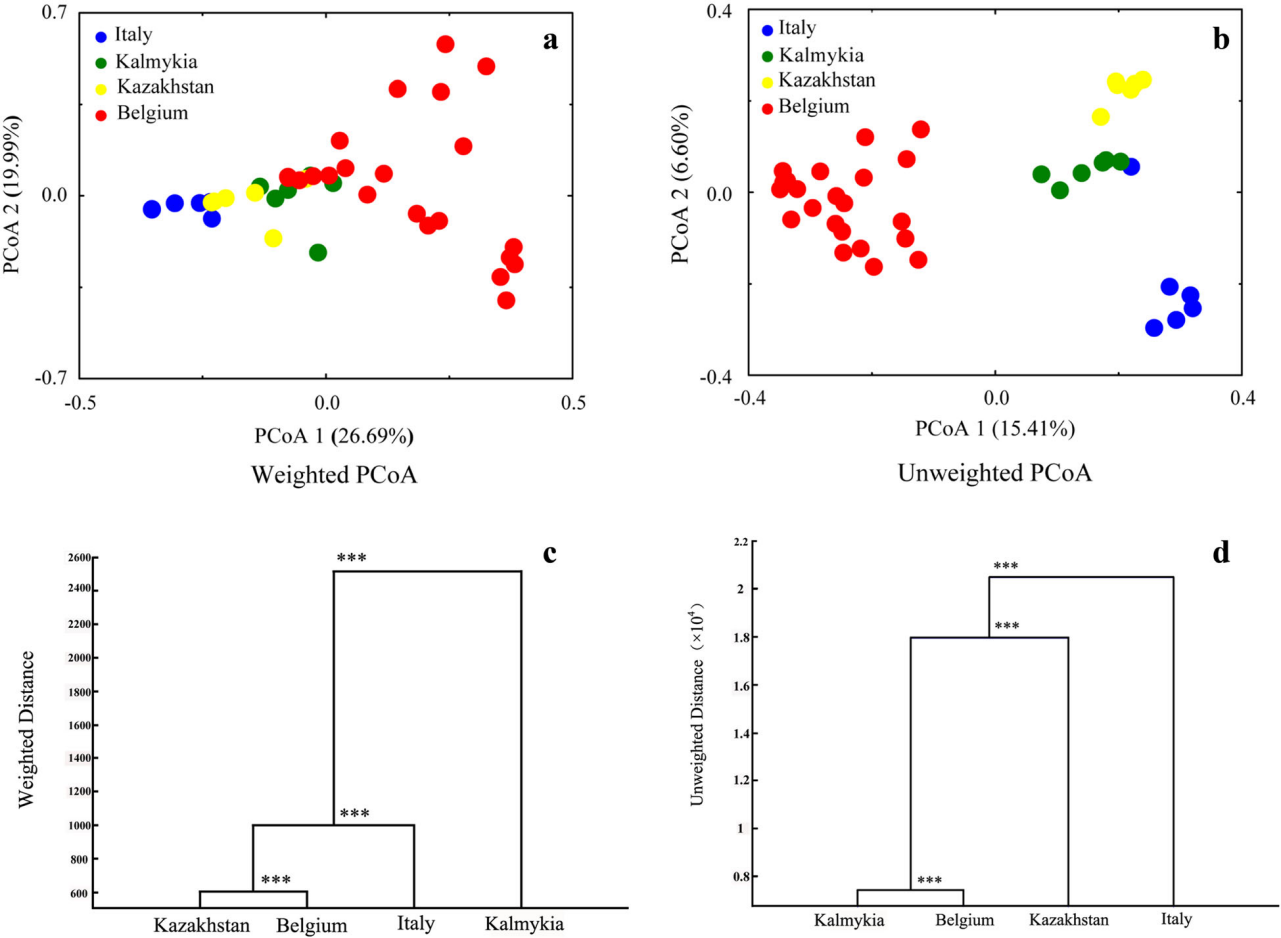
Comparison of the bacterial community structure of samples from the four countries Principal coordinate analysis based on the weighted (**a**) and unweighted (**b**) UniFrac distances (*blue = Italy*, *green = Kalmykia*, *yellow = Kazakhstan*, and *red = Belgium*). The cluster analysis was performed based on the weighted (**c**) and unweighted (**d**) Mahalanobis distances calculated by MANOVA of cheese bacteria communities, “***” represents *p* < 0.001

Then, PCoA based on the weighted (principal coordinate 1 and 2 accounted for 26.69 and 19.99% of the total variance, respectively) (Fig. 3a) and unweighted (accounted for 15.41 and 6.60% of the total variance, respectively) (Fig. 3b) UniFrac metrics revealed the existence of bacterial structural difference. On the unweighted PCoA score plot, clear clustering pattern based on the cheese origin was observed, while only a mild overlapping occurred on the weighted PCoA score plot, suggesting that the geographic location and origin of the cheese may be related to the distinct bacterial microbiotia composition. These results were further supported by MANOVA (*p* < 0.001) (Fig. 3c, d).

### Comparative analysis of the bacterial composition of the cheese samples

**Fig. 4:**
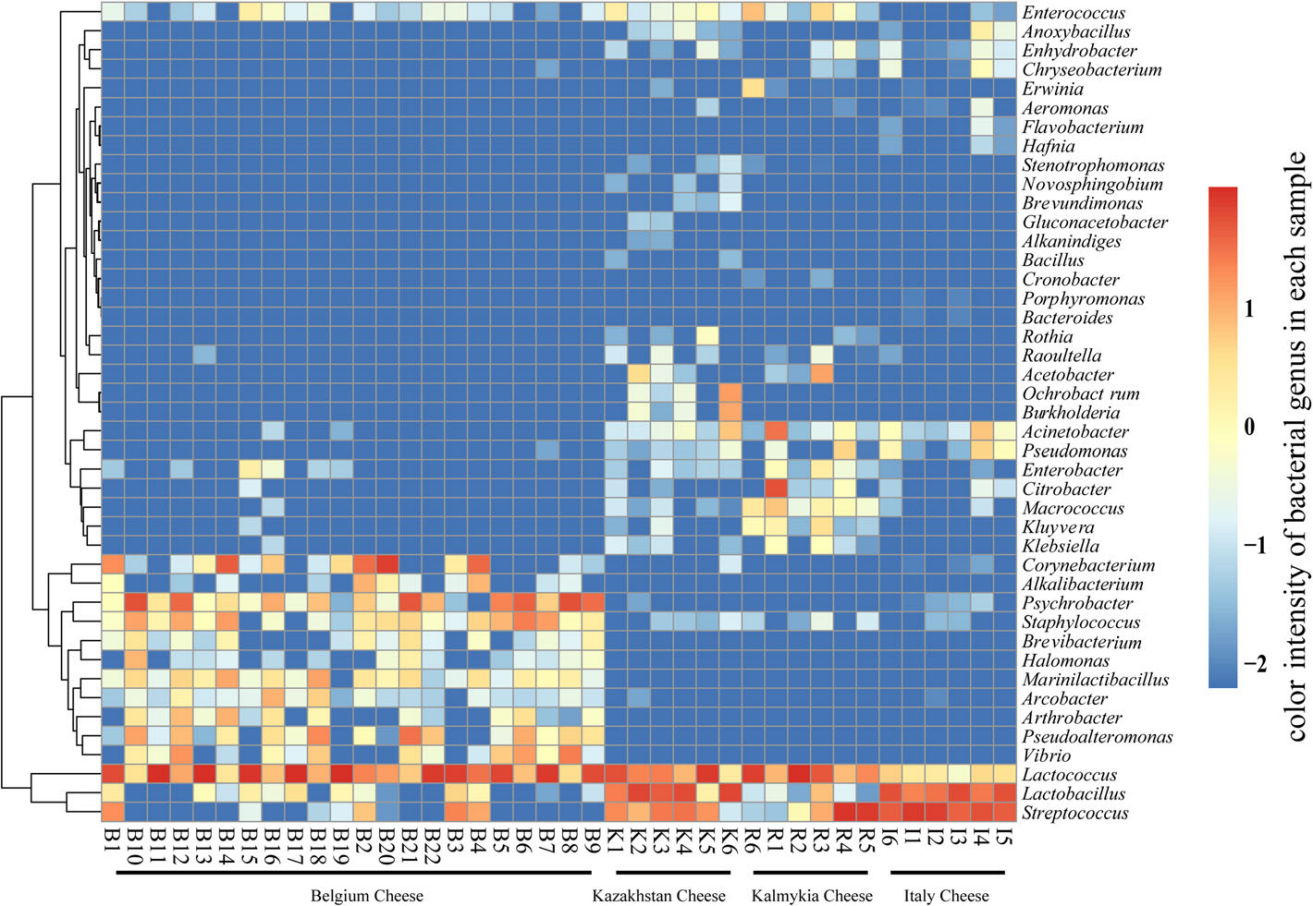
Heatmap depicting distribution of significantly different genera with *p* < 0.05 across groups Bacterial relative abundances are illustrated by the color scale

The proportions of the three most prevalent bacterial genera in the cheese samples from Kazakhstan, Belgium, Kalmykia and Italy were different, as shown in Table 2. In addition, the difference in bacterial composition of the 40 cheese samples was further evaluated by Kruskal-Wallis test *p* < 0.05 (Additional file 2: Table S4). The relative abundances of 43 genera were significantly different across groups. A heat map showing the distribution of these differential genera is presented in Fig. 4.

**Table 2:** The major bacterial genera in the cheese 16S rRNA datasets

Samples	Bacterial genera	Relative abundances (%)
Kazakhstan cheese	*Lactobacillus*	42.12
	*Lactococcus*	31.07
	*Streptococcus*	16.98
Belgium cheese	*Lactococcus*	44.32
	*Psychrobacter*	14.72
	*Corynebacterium*	9.80
Kalmykia cheese	*Lactococcus*	42.49
	*Streptococcus*	28.79
	*Citrobacter*	8.88
Italy cheese	*Streptococcus*	53.97
	*Lactobacillus*	39.2
	*Lactococcus*	2.88

**Fig. 5:**
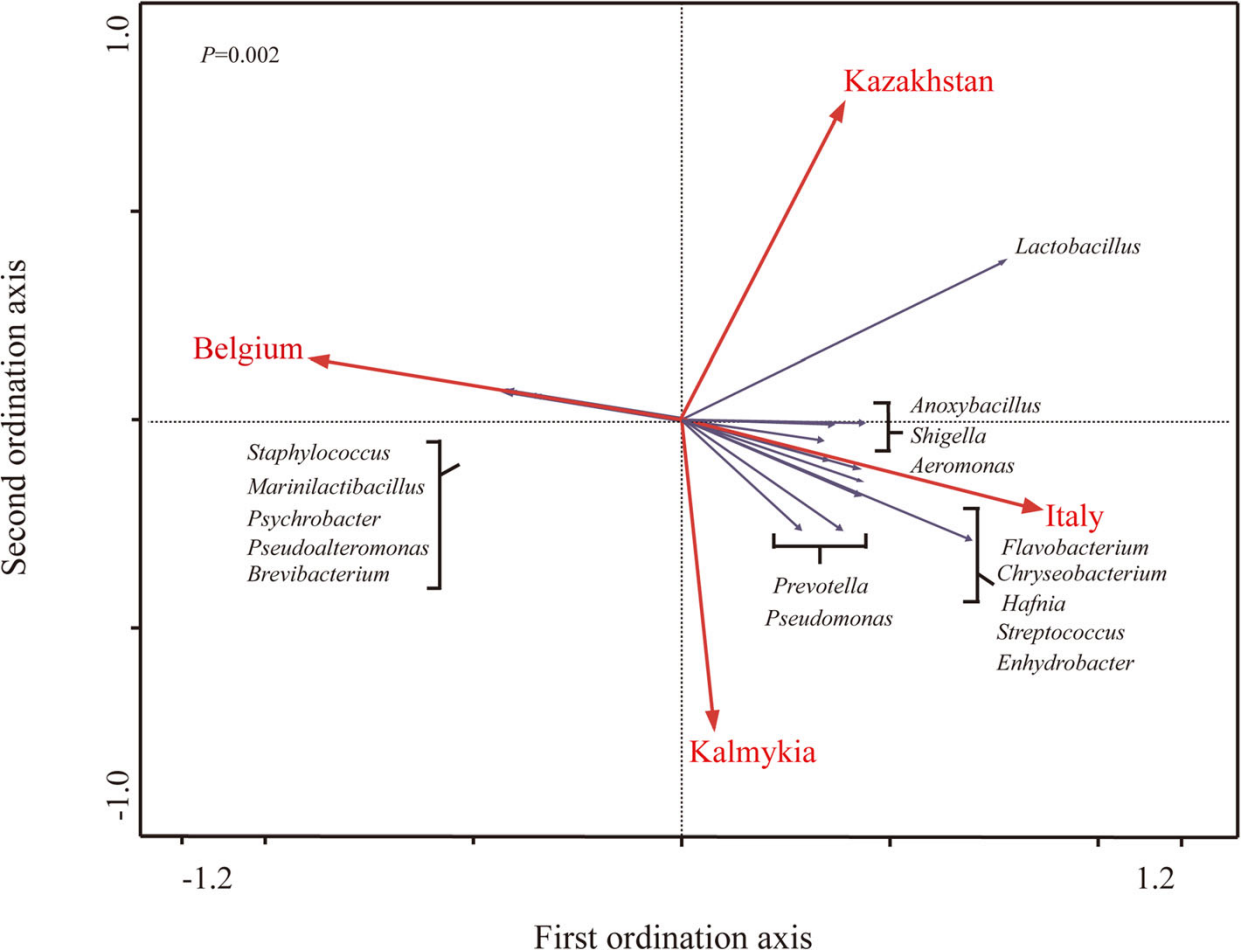
Biplot of redundancy analysis (RDA) of the cheese bacterial communities from four countries *Red* arrows represent the constrained explanatory variables, Kazahstan, Italy, Kalmykia and Belgium. Grey lines represent the response variables with the first ordination axis explaining for at least 35% and 16 genera of the variability of the bacterial microbiota. The *p*-value generated from the Monte Carlo permutation test is shown in the plot

Redundancy analysis (RDA) was performed to further identify the relationship between the sample geographical origins and the key responding OTUs that contributed to the microbial community structural difference (Fig. 5). The significant difference in the bacterial composition among the 40 samples was confirmed by a Monte Carlo permutation procedure (MCPP) (*p* = 0.002). Sixteen key genera were identified, of which at least 16% of the variability in their values was explained by the canonical axis. Eleven responsive genera were key genera in samples from Kazakhstan, Italy and Kalmykia. The other five genera (*Staphylococcus*, *Marinilactibacillus*, *Psychrobacter*, *Pseudoalteromonas* and *Brevibacterium*) were located at the left side of the ordination plot, suggesting that they were enriched in the Belgium cheese (Additional file 2: Table S5).

## Discussion

Our study used the SMRT sequencing approach to describe the Kazakhstan cheese bacterial diversity and revealed the unique characteristics of the microbiota structure of the artisanal cheeses in depth. The high-throughput sequencing of 16S rRNA gene has become a vital tool for identifying members of microbial communities present in traditionally fermented dairy products [36–38]. Studies of natural bacterial communities utilizing pyrosequencing have been limited to the identification of community composition to the genus level. This study further supports that the third generation PacBio SMRT is advantageous over the more traditional second generation next sequencing technology in describing microbiota communities in dairy products like cheese because of its capacity in generating longer reads. Using the current approach, bacterial profiles at a higher taxonomic resolution to the genus and even species level can be obtained without much difficulty.

Our results show that Firmicutes and Proteobacteria are the major phyla present in traditional artisanal cheeses. This result is in line with previous studies [13, 17, 39]. As the predominant species in the artisanal cheeses, *Lactococcus lactis* is commonly used as a starter during the manufacturing process because it has rapid acidification and curd production capacities [40, 41]. *Lactococcus lactis* also significantly contributes to the organoleptic properties and microbial quality of cheese by facilitating the acidification (the formation of lactic acid) [42, 43], aroma production (the degradation of casein), nisin synthesis, and enhancement of flavor and texture characteristics. *Lactobacillus helveticus* is another prevalent species found in our samples; this species is also a common starter that is used for cheese fermentation [44–46]. *Lactobacillus helveticus* is well-known for its proteolytic activity, which is of importance in reducing the cheese bitterness [47]. Furthermore, the proteolytic activity encoded by the proteolytic system of some *L. helveticus* strains is considered to be important in releasing antihypertensive peptides [48].

*Streptococcus thermophilus* and *Lactobacillus bulgaricus* are complimentary to each other and are essential for optimizing the milk fermentation environment during yoghurt and cheese making [49]. During the fermentation process, *S. thermophilus* supports the growth of *L. bulgaricus* by producing acid and utilizes the dissolved oxygen to produce CO_2,_ and creates the anaerobic conditions for the *L. bulgaricus* growth in milk. On the other hand, *L. bulgaricus* possesses more proteolytic enzymes to release amino acids, and then stimulates the growth of *S. thermophilus* [50, 51].

*Ochrobactrum lupine* was initially isolated from soil [52, 53]. Not many previous reports have identified members of this genus in cheese, but interestingly they were identified in the Kazakhstan cheese samples (K2, K4 and K6). *Acinetobacter baumannii* is a potential pathogen that may cause nosocomial infections [54]; and it is resistant to many clinically used antibiotics [55]. The presence of 16S rDNA amplicons representing microbes of environmental or clinical concerns might have been directly related to the cheese production environment and hygienic condition. Alternatively, such contaminants may be present in the air, soil and water, which could enter the raw materials or along the food processing procedure. It has been suggested that parallel negative controls should be included in this kind of microbiota profiling experiments, as the contamination might also source from the molecular biology grade water, PCR reagents, and DNA extraction kits that are used in the experiment [56]. For example, the *Ochrobactrum* genus has been reported to be one of those contaminants originated from laboratory reagents. Since our study did not include any parallel negative control; further study will be needed to confirm its source.

In order to further understand the characteristics of Kazakhstan cheese, we extracted 16S rRNA gene nucleotide sequences of traditionally artisanal cheese of three other regions, including Belgium, Italy and Kalmykia, from public gene databases and performed comparative analysis with the Kazakhstan cheeses collected in this study. Zhong et al. [57] comparatively analyzed the naturally fermented milk microbiota profiles based on different 16S rRNA gene regions. However, such analysis was limited by the low number of public accessible datasets of cheese 16S rRNA data, particularly full length gene sequence [58]. Based on multivariate statistical analysis, the artisanal cheese of Kazakhstan has its own characteristics. There were apparent differences in the microbiota communities between cheeses of different groups, as revealed by PCoA and MANOVA, suggesting that the geographic location may play a role in shaping the sample microbiota. Similar geographical-based variations were reported in traditional fermented vegetables [59] and home-made fermented milks [39, 44, 60]. By comparing with the cheese production methods, the four kinds of cheese varied greatly in their ripening stage (Table 1). The heatmap shows apparent differences between the microbiota composition of the Belgium cheese from the others. The type of milk might also have contributed to the differences in the microbiota composition after fermentation due to both the intrinsic nutrient components and the pre-existing natural starter cultures. Buffalo milk has a higher fat and protein content than cow milk [61], and thus water-soluble and non-protein-nitrogen. The analyses of the microbiota of raw milk, nature whey and water buffalo mozzarella cheese by high-throughput sequencing indicated that nature whey had an important effect on the final microbial composition of cheese [14, 62]. Thus, apart from the geographic location, other factors including origin and type of cheese, cheese-making technology, processing and environmental conditions may together shape the ultimate bacterial community profile [63–65]. After all, our study has provided novel and interesting information regarding the bacterial microbiota of local Kazakhstan cheeses.

## Conclusion

To sum up, the current study analyzed the bacterial communities of six traditional Kazakhstan cheese samples by SMRT. A total of 140 genera from 14 phyla were identified from the samples, with the predominant species of *Lactococcus lactis* (28.93%), *Lactobacillus helveticus* (26.43%), *Streptococcus thermophilus* (12.18%) and *Lactobacillus delbrueckii* (12.15%). By comparatively analyzing the 16S rRNA gene sequences generated from this study and those retrieved from public databases, unique bacterial community signatures could be identified in the Kazakhstan cheeses, which were largely different from those of Belgium, Kalmykia and Italy. Thus, the cheese origin does seem to play a role in shaping the sample microbiota composition.

## Additional files

Additional file 1: Figure S1. The appearance of the six Kazakhstan traditional artisanal cheeses. (TIF 5836 kb)
Additional file 2: Table S1. The nutritional information of Kazakhstan cheeses (the content of 100 g sample). **Table S2.** Number of sequences and OTUs, microbial diversity and richness indexes of Kazakhstan cheese samples. **Table S3.** Relative abundances of bacterial phyla in the six Kazakhstan cheese samples. **Table S4.** Average relative abundances of the 43 main bacterial genera across the four sample groups. **Table S5.** Relative abundances of 16 key bacterial genera across the four sample groups. (XLS 73 kb)
Additional file 3: Figure S2. Rarefaction (left) and Shannon diversity (right) curves of samples. Each line represents the dataset of one sample. (TIF 8495 kb)
