# Supplementary figures and images for: Bacterial microbiota of Kazakhstan cheese revealed by single molecule real time (SMRT) sequencing and its comparison with Belgian, Kalmykian and Italian artisanal cheeses
Source: BMC Microbiol. 2017 Jan 9;17:13. doi: 10.1186/s12866-016-0911-4 (PMC5223556; doi:10.1186/s12866-016-0911-4)

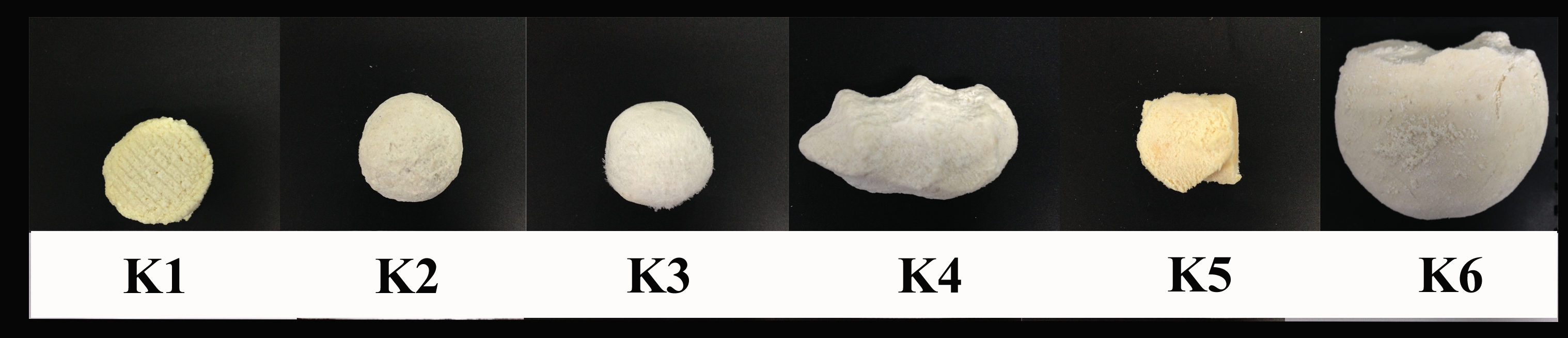

Supplement: Additional file 1: Figure S1. — The appearance of the six Kazakhstan traditional artisanal cheeses. (TIF 5836 kb) [file 12866_2016_911_MOESM1_ESM.tif]
